# Athletes’ experiences of using a self-directed psychological support, the BAck iN the Game (BANG) smartphone application, during rehabilitation for return to sports following anterior cruciate ligament reconstruction

**DOI:** 10.1186/s13102-023-00731-2

**Published:** 2023-09-19

**Authors:** Magnus Ringberg, Ann Catrine Eldh, Clare L Ardern, Joanna Kvist

**Affiliations:** 1 Unit of Physiotheray, Department of Health, Medicine and Caring Science, Linköping University, Linköping, SE-581 83 Sweden; 2 Department of Public Health and Caring Sciences, Uppsala University, Box 564, Uppsala, SE-751 22 Sweden; 3 Division of Nursing Sciences and Reproductive Health, Department of Health, Medicine and Caring Science, Linköping University, Linköping, Sweden; 4 Department of Physical Therapy, University of British Columbia, Vancouver, British Columbia V5Z 1M9 Canada; 5 Sport and Exercise Medicine Research Centre, La Trobe University, Melbourne, VIC 3086 Australia; 6 Stockholm Sports Trauma Research Center, Dept of Molecular Medicine & Surgery, Karolinska Institute, Stockholm, SE-171 77 Sweden

**Keywords:** Anterior cruciate ligament reconstruction, Athletes, eHealth, Mobile phone, Psychological support, Rehabilitation, Return to sports, Sports injury

## Abstract

**Background:**

Following anterior cruciate ligament reconstruction (ACLR), many athletes do not return to their sport, often driven by concerns about re-injury. Psychological support strategies might help, but are not routinely included in rehabilitation after ACLR. The BAck iN the Game (BANG) intervention is a 24-week eHealth program delivered via smartphone application (app), beginning directly after ACLR, with a self-directed approach that aims to target the specific challenges athletes encounter in rehabilitation.

**Aim:**

To describe athletes’ experiences of using the BANG app during rehabilitation, to support returning to sport following ACLR.

**Method:**

Participants were athletes, in contact and/or non-contact pivoting sports, who had ACLR with the goal to return to sports. Semi-structured, individual interviews were conducted 6–10 months after their ACLR; all had access to the BANG intervention. Verbatim transcripts were analysed with a qualitative content analysis.

**Results:**

The 19 participants were 17–30 years, mean 21.6 years (SD 3.5); 7 men and 12 women. The analysis generated three main categories. (A) *Interacting with the app* illustrated how, when, or why the participants engaged with the app. The app was helpful because of its varying content, the notifications served as reminders and participants stopped using the app when no longer needing it. (B) *Challenging experiences with the app* illustrated that the app itself came with some difficulties e.g., content not appearing with the right timing and material not tailored to their sport. (C) *Supportive experiences with the app* reflected how the app facilitated the participants’ rehabilitation progress; it included positive aspects of the app content and navigation, boosting their confidence to return to sport, and motivated them to continue with rehabilitation.

**Conclusion:**

The analysis of the interviews illustrates athletes’ awareness in interacting with, and the challenging and supportive experiences of using the app. The BANG app might provide support for returning to sport, primarily psychological support, as an adjunct to regular physiotherapy-guided rehabilitation. Athletes’ experiences of the BANG app could be improved by healthcare professionals providing additional advice about when to use which content and why.

**Trial registration:**

ClinicalTrials.gov, NCT03959215. Registered 22 May 2019.

**Supplementary Information:**

The online version contains supplementary material available at 10.1186/s13102-023-00731-2.

## Background

One of the most serious sports-related knee injuries is a rupture of the anterior cruciate ligament (ACL), which typically requires patients to engage in musculoskeletal rehabilitation to restore knee function [1]. Athletes in cutting and pivoting sports usually undergo surgery with reconstruction of the ACL (ACLR) aiming to stabilize the knee joint and help the athlete return to sports [2]. Yet, athletes of all ages and activity levels can have problems with returning to sport after ACLR [3, 4]. Ardern et al. concluded that 4 in 5 people return to sports after ACLR, but only half to their preinjury competitive sport level [3]. After ACLR, athletes can feel uncertain about making a full recovery [5] and fear has consistently been reported among individuals following ACLR [6–9]. Greater psychological readiness, a construct that incorporates confidence, emotions, and risk appraisal, is associated with a greater likelihood of returning to preinjury sport [10]. Many psychological factors including confidence, anxiety, and risk appraisal are potentially modifiable [11]. Patients who report poor knee self-efficacy, kinesiophobia, and fear avoidance following the acute stage of rehabilitation may benefit from targeted interventions to improve these psychological constructs [12].

With the goal of returning to sport, the focus of rehabilitation has traditionally been on recovering physical attributes [13]. Yet, more recent best practice guidelines for musculoskeletal injury rehabilitation include recommendations to address psychological factors [14] and today, consensus in return to sports suggests a biopsychosocial perspective to prepare athletes for return to play [15]. Athletes desire psychological support for their return to sport, but physiotherapists deem themselves ill-equipped to deliver adequate psychological support during ACL-injury rehabilitation [16]. Using psychological support delivered with video-, website- or telephone- interventions to complement face-to-face ACL-injury rehabilitation is of growing research interest [17–19].

To meet the demand of a psychological support in ACL-injury rehabilitation, we designed an eHealth intervention called BAck iN the Game (BANG), to be available on-demand and delivered via a smartphone application (app) [20, 21]. While some studies have described the experience of rehabilitation after ACLR from the injured athlete’s perspective [6, 7, 22–26], there are no reports of participants’ experiences when using an app for self-directed psychological support that is designed to complement usual rehabilitation after ACLR. In any development of a complex intervention from idea to implementation, there is a need to understand the end-user’s perspective [20, 27, 28], in this case, athletes with ACLR who use an app to support their psychological readiness to return to sport. Therefore, this paper describes athletes’ experiences of using a self-directed psychological support, the BAck iN the Game (BANG) smartphone application, during rehabilitation to support returning to sport following ACL reconstruction.

## Method

### Design

This paper presents an interview study analysed with a qualitative content analysis, employing an inductive manifest approach [29, 30]. Qualitative content analysis is used to understand the complexity of people’s experiences [31]. An inductive approach, considering the manifest content of the dataset, is appropriate when prior knowledge regarding the phenomenon under investigation is limited or fragmented [29]. In the inductive approach, codes and categories (or themes) originate from the data [30], most often semi-structured interviews with the purpose of representing participants’ experiences [32]. The study is reported according to the consolidated criteria for a transparent report of qualitative research (COREQ) guidelines [33].

### Research settings -the BANG intervention and trial

The BANG intervention is a 24-week eHealth program, beginning directly after ACLR, with a self-directed approach that aims to target the specific psychological challenges encountered by the individual, such as confidence for return to sport. The intervention has been described previously [20, 21]. In brief, it comprises seven modules that complement usual rehabilitation: (1) goal setting, (2) confidence for recovery, (3) confidence for return to sport, (4) confidence for performance, (5) confidence to stay injury-free, (6) support to handle thoughts and emotions related to recovery and return to sport, and (7) education about knee injury, recovery, return to sport, and safe sports participation. The program stands alone from the usual physiotherapy treatment and serves as an additional support for the individual throughout the rehabilitation.

The BANG trial is a randomised controlled trial testing the effectiveness of the BANG intervention [20, 21]. Participants are recruited before they undergo a scheduled ACLR and are randomised to an experimental and a control group. The inclusion criteria are listed in Table 1. Each participant in the experimental group receives 40 notifications, plus additional reminders as notifications and/or short message (SMS) with a link to app content and task to respond with, over the course of the 24-week intervention. Participants can re-visit previous app material at any time up to 24 months post ACLR. Additional questionnaires are also sent from the app at specific timepoints throughout the intervention to collect primary and secondary outcomes for the BANG trial.

### Participants

For this study, potential participants were identified from the experimental group of the ongoing BANG trial [20, 21]. Participants were purposefully recruited at six to ten months after ACLR. A list of potential participants was drawn based on time from surgery when the interviews were planned to be conducted. We used maximum variation sampling to include different sport participation, both sexes, different timepoints from ACLR to the interview and different response rates to the BANG intervention tasks (notifications). With two participants declining and two who could not be reached, there were 19 individuals who were interviewed. The participants were contacted via SMS with information about the interview and confirmation that their participation was voluntary.

Individual semi-structured interviews were conducted via telephone between October 2020 and June 2021 by the same interviewer (JK) and no interview was repeated. An interview guide (Appendix 1) was used as a foundation for the interviews and consisted of open-ended questions encouraging participants to describe their experiences. The interview guide originated from the previous feasibility study [20], and was refined by an experienced psychologist in eHealth and two of the authors (JK and CA). Questions in the interview guide aimed to capture participants’ experiences of the BANG app and their experiences of using it during rehabilitation after ACLR. All interviews were audio recorded and transcribed verbatim by a professional transcriber. The transcripts were not returned to the participants for comments, and the participants were not asked to provide feedback on the findings. None of the authors had any relationship with the participants.

**Table 1:** Participant inclusion and exclusion criteria for the BANG trial

Inclusion criteria	Exclusion criteria
Age 15 to 30 years at the time of the ACL injury	Medial or lateral collateral ligament injury requiring surgery
Unilateral primary ACL rupture (diagnosed by clinical examination and/or MRI)	Posterior cruciate ligament injury
< 12 months between the injury and ACLR	Meniscus injury and/or treatment requiring alteration to usual rehabilitation care
Performing contact pivoting or non-contact pivoting sport at least twice per week prior to the ACL injury	Articular cartilage injury and/or treatment requiring alteration to usual rehabilitation care,
Intention to return to sport following ACLR	Previous ACL injury to either knee
Normal/healthy contralateral knee	Injury to either lower limb that required medical care during the 12 months prior to index ACL injury
Fluent in written and spoken Swedish language	Other injury or illness that could affect knee rehabilitation or taking medication for mental health problems

### Data analysis

The research team consisted of two professors (AE, JK), one senior researcher (CA), and one PhD student (MR). Three of the researchers are female (AE, CA, JK), and one male (MR). One of the authors has extensive experience in qualitative research (AE), and two have extensive experience in ACL injuries and return to sport. Three are physiotherapists (MR, CA, JK) and one is a registered nurse (AE).

The transcribed text from the interviews was analysed using an inductive content analysis, as described by Elo and Kyngäs [29]. Microsoft Excel was used to manage data. First, to obtain a sense of the whole and to ensure transcript accuracy, the transcripts were read and re-read several times by two authors (MR, JK) independently. Second, we identified and coded meaning units that corresponded to the study aim. Next, the extracted codes were analysed: codes with the same or similar meaning were merged, considering the essence of each meaning unit, and subcategories were formed. The subcategories were then abstracted to categories, providing for a more advanced account of the participants’ experiences. The subcategories and categories were appraised multiple times, in a critical discourse between authors (MR, AE, JK) until consensus was reached. The final analysis was discussed with a fourth author (CA) to affirm a transparent and trustworthy report of the findings.

### Ethical considerations

For the interviews, the participants were informed about the voluntary nature of their participation, the measures to ensure confidentiality, and that their consent to participate could be withdrawn at any time (without needing to provide a reason). Written consent was obtained from all participants in the BANG trial and oral informed consent was obtained from all participants prior to this qualitative study. Personal data were stored on a secure university server (for electronic data). The material was coded and presented in an anonymized way to ensure that individuals could not be identified. This study was approved by the Swedish Ethical Review authority, Regional Ethical committee in Linkoping, Sweden (D.nr. 2018/45 − 31).

## Results

The 19 participants were aged between 17 and 30 years, mean age 21.6 years (SD 3.5). There were 7 men and 12 women; all were performing contact or non-contact pivoting sport prior to their ACL injury. The participants played football (*n* = 8), handball (*n* = 7), both football and handball (*n* = 1), floorball (*n* = 1), American football (*n* = 1), or track and field (*n* = 1), respectively. Three of the participants were national-level athletes, the other 16 participated at sub-elite level. Interviews were on average 17 min long (range 7–33 min) and the transcripts contained a mean of 11 068 characters without spaces (range 5062–24,544 characters).

**Fig. 1:**
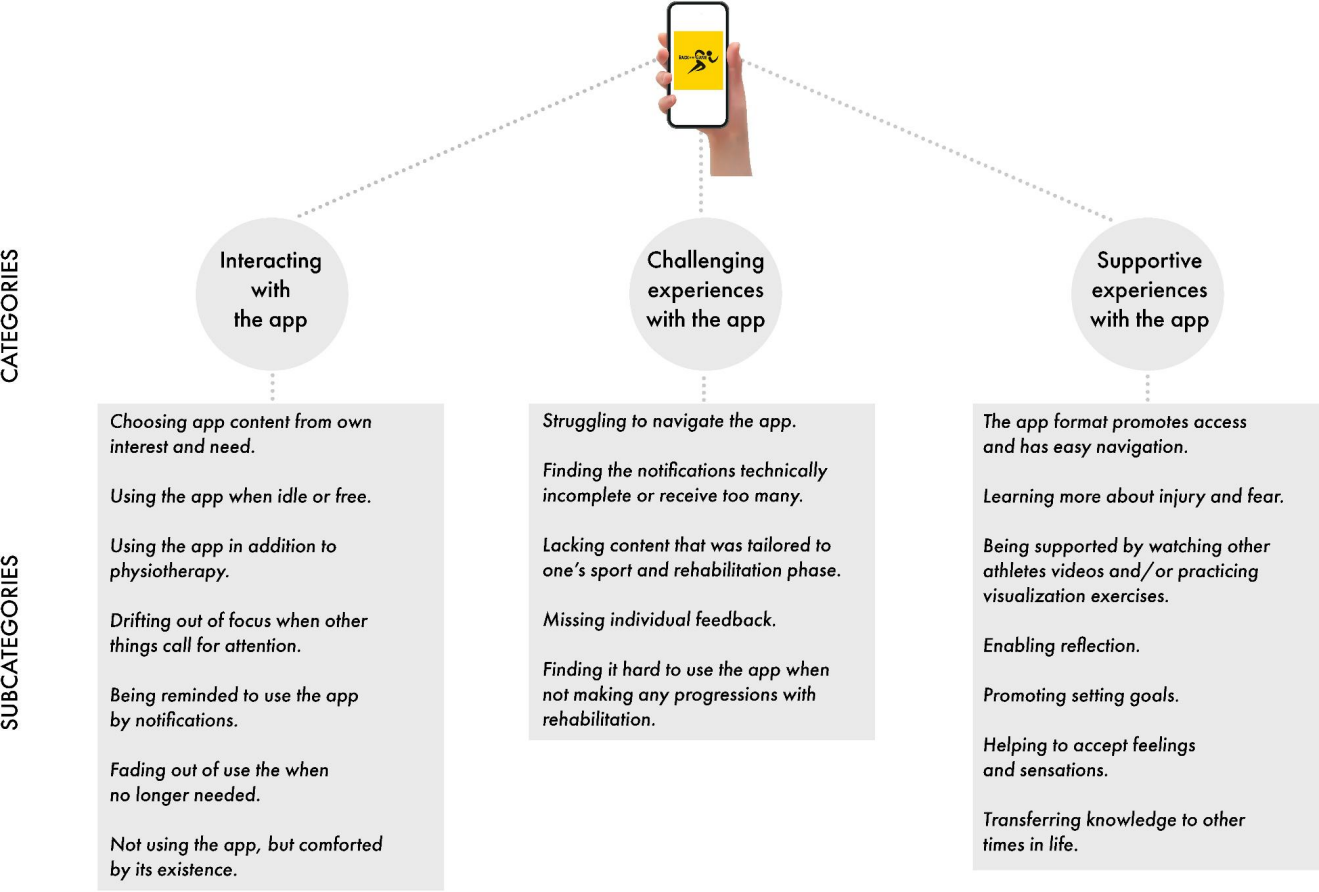
Overview of the athletes´ experiences of using the BANG app during rehabilitation following ACL reconstruction

The qualitative content analysis generated three main categories, with 19 associated subcategories. The categories and subcategories are described in text below and illustrated in Fig. 1.

### Interacting with the app

The category *Interacting with the app* refers to how, when, or why the participants were engaged with the app, and consists of seven subcategories. *Choosing app content from own interest and need* illustrates how participants allowed themselves to use specific content of interest or repeated helpful parts of the material. This included, choosing to revisit a video, practicing self-reflection exercises, or listening to relaxation audio. *Using the app when idle or free* illustrates that the participants chose to use the app when it suited them; they mainly used the app early in rehabilitation—when on sick leave and sedentary. Participants used the app when they felt like they had the time and interest throughout the entire intervention period.

*It was great to have a psychological support. In the app menu I was able to choose based on my own needs. (ID 2)*

*I used the app especially in the beginning of my rehab; when I was at home and on sick leave. (ID 3)*

*Using the app in addition to physiotherapy* illustrates that the participants perceived the app as a resource for psychological support, promoting progression and complementing physiotherapy sessions. *Drifting out of focus when other things call for attention* describes that the participants stopped using the app when other aspects of life took precedence (e.g., work or school). *Being reminded to use the app by notifications* suggests that reminders helped participants to engage with the content; without notifications participants would have forgotten to use the app.

*I think the app was a good extra support in my rehabilitation, to ensure I got as much out of it as possible. (ID 4)*

*In my busy life, I prioritize other things than the app. (ID 15)*

*I used the app when I received the notifications. (ID 5)*

*Fading out of use when no longer needed*, refers to how participants’ use of the app stopped for reasons such as no longer needing it or one’s needs have been met. Participants described being mainly occupied with thoughts and worries about the injured knee-joint early in rehabilitation and felt supported by the app content at the time. As rehabilitation progressed, the app was not considered necessary to succeed. *Not using the app, but comforted by its existence*, represents how the participants shared that they managed their rehabilitation without the app; the participants valued the app, and its availability ‘on-demand’. Further on, the app was not considered necessary to succeed in rehabilitation.

*I used the app in the beginning of my rehabilitation, but after a while I didn’t need it any more. (ID 12)*

*I didn’t need the support from the app, but it was nice to have. It confirmed the next phase of my rehabilitation. (ID 19)*

### Challenging experiences with the app

The category *Challenging experiences with the app* depicts the difficulties participants experienced with the BANG app, and encompasses five subcategories. *Struggling to navigate the app* primarily describes that the participants could not easily navigate the goal setting content because it was not easy to locate when navigating the app. *Finding the notifications technically incomplete or receiving too many* describes how the participants experienced technical problems and did not receive notifications about new app material. Participants described that notifications were delivered in the beginning, but if they changed their cellphone, they did not receive further notifications. At times, they experienced too many notifications, which was particularly stressful if they were in school or busy with other tasks.

*I couldn’t locate everything in the app. (ID 7)*

*I was annoyed when I received notifications and didn’t have the time to engage with the app. (ID 9)*

*Lacking content that was tailored to one’s sport and rehabilitation phase* refers to how the participants had identified a lack of alignment between the app material and their own timeline post-ACLR; they felt the content was either too early or too late. When the app content did not appear with the right timing, it was difficult to engage with the material. Participants also described that the app lacked material that was tailored to one’s own sport, and they couldn’t relate to some predefined multiple-choice alternatives in the app or to watching videos with athletes in sports other than their own. Breathing and relaxation exercises in the app were perceived as difficult and challenging to engage in.

*It was difficult to relate to other types of sports than mine. (ID 1)*

In *Missing individual feedback*, participants wished that someone could have read their responses from surveys, providing individual feedback. Participants felt that the app would have helped them more if it included personalized options in ready-made questions. Further, the participants described *Finding it hard to use the app when not making any progressions with rehabilitation*: it was less motivating to use the app during a plateau of progression or setbacks, losing their incentives to engage with the app.

*I didn’t receive any feedback on my goal setting in the app. (ID 18)*

*Early in my rehabilitation I was really struggling. I couldn’t get anywhere with my physical exercises; that was a difficult time for me to use the app. (ID 8)*

### Supportive experiences with the app

The category *Supportive experiences with the app* reflects how the app facilitated the participants’ rehabilitation progress; it encompasses seven subcategories, including positive aspects of the app content and navigation, boosting their confidence to return to sport, and motivation to continue with rehabilitation. *The app format promotes access and has easy navigation* highlights how participants appreciated the accessibility of the app and that the navigation was intuitive: simple to use, logical and with a pedagogy to the content. The app did not require a lot of writing for the participants as most app content had ready-made answers and the menu was easy to navigate. *Learning more about injury and fear* represents that the participants gained knowledge from the app content; the app became a helpful extra information resource, in addition to the doctor and the physiotherapist.

*To have access to an app is much better than a website. (ID 14)*

*Besides the information I receive from my doctor and physiotherapist, it’s nice to have the extra tutoring [that comes with the app]. (ID 16)*

The educational content of the app helped participants understand the process of being in rehabilitation; it was comforting to read and watch (text and videos) that fear is common during return to sport. Participants described that *Being supported by watching other athlete’s videos and/or practicing visualization exercises* assisted them in knowing that others have had similar injuries and experiences. Learning about other athletes’ ups and downs in rehabilitation offered a realistic expectation for their own experience. Knowing that obstacles can occur in or during rehabilitation validated their worries about setbacks, as did knowing that a loss of motivation can also happen to others. When other athletes shared (via the app content) how they began their rehabilitation post-surgery on the couch and then later returned to sport, participants were inspired: they described how the stories gave hope that they too would succeed. Further, practicing visualization exercises was supportive to prepare for upcoming movement skills in their rehabilitation.

*It was a learning experience watching other athletes talking about the same type av injury as I have. (ID 6)*

The subcategory *Enabling reflection*, illustrated an inspiration to sit down, and think about how one feels in a particular moment. The participants described how the app helped them to reflect about returning to sport and whether they trusted their injured knee. While reflection could be scary, it helped maintain motivation. Yet, if the exercises with reflections occurred too often in the app, with not enough time in between, it was less helpful: one needed to have made some progress before one could take on the next perspective. Using the app for reflections was particularly helpful due to its format: the participant was safe and undisturbed in their private space.

*Reflections are helpful; I get a chance to ask myself ‘how do I really feel’? (ID 17)*

*Promoting setting goals* refers to the act of phrasing one’s ambition when it comes to rehabilitation and returning to sports: the app helped with a selection of goals to choose from. With the benefit of hindsight, participants could go back to their previous goals and track their rehabilitation progression, regardless of how small the steps were. The app was *Helping to accept feelings and sensations*, and participants were supported in terms of self-compassion and body awareness. Participants described that during setbacks in their rehabilitation, the app helped them navigate feeling low; the app reinforced that it is okay to have such feelings. When being encouraged not to suppress feeling low, a sense of normalization could appear, and participants felt less lonely in their rehabilitation. Exercises with breathing and relaxation helped to access body awareness. *Transferring knowledge to other times in life*, illustrates that the participants learned of such aspects from the app, and implemented them in other life situations not related to sports.

*Setting goals has helped me to stay focused on what’s most important in my rehabilitation. (ID 10)*

*The app made me aware of my knee and my fear. It helped me to understand more about my feelings and how to accept them. (ID 11)*

*I think I would use the experience from the app if I would have a new injury. (ID 13)*

## Discussion

Psychological factors can hinder full recovery after ACLR. Individuals in rehabilitation and health professionals (such as physiotherapists) have expressed a need for psychological support during rehabilitation [16, 23, 24]. In the present study, participants in a randomized controlled trial of an eHealth intervention that aims to increase confidence for return to sports, shared their experiences of the BANG app and of using it during rehabilitation after ACLR. The analysis of the interviews illustrates participants’ awareness in interacting with, and challenging and supporting experiences of the app. With the BANG app, we wanted to learn how far one could get with self-directed care through a more generic eHealth intervention that was feasible to deliver to a range of participants with ACLR. Most importantly, we found that the athletes’ attentiveness for the app varied, but overall, the app provided additional support to that acquired from and within the rehabilitation process. Yet, the usefulness of the app was associated with both *if* and *how* the app corresponded to their individual needs and their progress in rehabilitation (or lack thereof).

In our analysis, the category *Interacting with the app*, and particularly the subcategory *Choosing app content from own interest and need*, illustrated how the participants chose and repeatedly employed cognitive-behavioral content from the app (using a task menu) during rehabilitation. In another intervention with cognitive-behavioral based physical therapy (CBPT) as psychological support, participants received seven sessions with controlled breathing, grounding, setting activity goals, monitoring self-talk, setting daily intentions, present-mindedness, managing setbacks, and guided imagery delivered using motivational interviewing by a physiotherapist. The intervention stopped eight weeks after ACLR and all participants completed all sessions [19]. With a one-to-one session, a therapist can direct the intervention and verify that each participant receives specific content and tailor the process. Our self-directed approach was different, and our results revealed that participants’ own interest and needs directed their involvement.

Participants shared how they forgot to use the app in their daily life and stopped using the app in a gradual manner (found in the sub-categories *Drifting out of focus when other things call for attention* and *Fading out of use when no longer needing it)*. Adherence is a problem in other interventions with psychological support after ACLR [18]. Lack of time has previously been described as the primary perceived barrier to adherence [34]. With the BANG app, we used notifications and SMS to communicate with the participants, which can improve adherence in self-directed programs [35–37]. Using an app as a platform for an intervention is convenient and promotes easy-access [38]. Still, participants expressed that in everyday life they were busy with other (private, school- or job-related) digital activities, which became a barrier to using the app.

Participants experienced that the BANG app provided psychological support and it had become an educational resource through rehabilitation (subcategory, *Using the app in addition to physiotherapy).* Physiotherapists report that they felt ill-equipped to deliver adequate support during ACL injury rehabilitation [16]. Therefore, an additional asset delivered via eHealth can be one alternative for delivering and receiving psychological support. It seems that digital support can complement face-to-face sports injury rehabilitation [18, 19] and future research will reveal how the BANG app is experienced by individuals when used in collaboration with the physiotherapist at selected time-points during rehabilitation. This may address the request for a more individualized support from the app (illustrated as *Missing individual feedback*).

A barrier for the participants was that they felt that *Notifications were technically incomplete or I receive too many*. At the same time, *Being reminded to use the app by notifications*, illuminated that the reminders from the app helped participants to engage with the content. Without such notifications, participants would have forgotten to use the app. Digital triggers such as text messages, emails, and push alerts are designed to focus an individual on a desired goal by prompting a reaction at the appropriate time [39]. Although eHealth intervention developers should carefully consider when and how to use notifications [39]. We also acknowledge that changing phone system (Android or iPhone) can disrupt and challenge technical settings for notifications.

*Missing individual feedback*, revealed that participants felt like they weren’t confirmed on a personal level. We acknowledge that some participants wanted more individual feedback throughout the 24-week intervention, and adding more individual communication (e.g., chat function in the app with a physiotherapist) could address this tailoring request. Another program with influences from psychology is the novel MOTor Imagery to Facilitate Sensorimotor Re-Learning (MOTIFS) model, which is delivered as individual sessions by a physiotherapist after traumatic knee injury [40]. Participants who received the MOTIFS treatment model described how they gained psychological support together with combined motor learning. Additionally, they perceived the program as preparing for return to activity and helping to cope with negative psychological factors during knee-injury rehabilitation [41]. The choice between a highly tailored psychological support or a one-size-fits-all intervention requires prioritising resources with accessibility; the BANG app primarily focuses on the large and diverse group of patients with ACLR.

For almost 20 years, psychological factors have been a common topic in ACL injury research [12, 13, 42–44]. Previous studies described how learning psychological strategies was essential to returning to sport following ACLR [23, 45]. In our study, participants expressed that they gained support from the app when dealing with thoughts about fear of reinjury (subcategory *Learning more about injury and fear).* For some participants, *Finding it hard to use the app when not making any progression with rehabilitation*, revealed that it was less motivating to use the app at certain times. Using a self-directed tool in collaboration with a physiotherapist at selected time-points throughout rehabilitation might avoid the less helpful experiences of using the app. Instead of an independent and self-directed app, there is potential for the BANG app content to become integrated and individualized with assistance from the physiotherapist. Previous research has shown that digital support can complement face-to-face sports injury rehabilitation [18, 19].

Participants appreciated using the app for reflections (subcategory *Enabling reflection*) as they were undisturbed in a private space. This indicates that a digital self-directed approach may be a favorable option to face-to-face treatment for some participants at some points in rehabilitation. Requiring and requesting psychological support can carry stigma [46], suggesting that an eHealth intervention might be less embarrassing to adopt than going for an appointment with a psychologist. Furthermore, the app transcends geography, boosting access to the whole rehabilitation team. The app is available at a low cost, thus bridging other barriers that might prevent athletes from accessing the psychological support that they need to return to sport after injury [20]. Harnessing eHealth for a psychological support that serves as a complement to ACL injury rehabilitation might become a helpful tool for clinicians. Using the BANG app, with usual ACLR care guidelines, for participants who need it or have an interest in psychological support, might represent an accommodating strategy in rehabilitation. Our results can inform future development of BANG and its implementation with relevant stakeholders in the clinical field of ACL injury rehabilitation.

### Limitations of this study

We used maximum variation sampling to include different timepoints from ACLR to the interview. Some participants answered early after the 24-week intervention and others up to four months after completing the intervention. There is potential for recall bias in the semi-structured interviews as the participants answered retrospectively about their experience of using the BANG app [47]. All participants were recommended to continue to use the app after the 24-week intervention, but no new material was released. It is possible that participants who continued to use the app had better recall of the content during the interviews. We deliberately chose different timepoints for the interviews (six to ten months after ACLR), to capture a wide range of experiences with the BANG app. We also purposefully recruited participants with different app usage, from different sports, and both sexes to capture a wide range of experiences with the BANG app. There were slightly more female participants enrolled in the study: male and female high school athletes have previously described different psychological factors related to return to sport and locus of control as well as psychological distress, which indicates that sex-specific psychological interventions to overcome psychological barriers after ACLR may be warranted [48]. In the BANG trial, there were slightly more females enrolled (54%), so the representation in this interview study is comparable with the RCT. We are also aware of selection bias, and participants who agreed twice (once for the BANG trial and again for the interview study) to participate in research might not reflect the typical experience of someone using an eHealth intervention in ACL injury rehabilitation. We did not interview people who withdrew from participating in the BANG trial. The perspectives of athletes who do not want psychological support must also be considered in future research, and included in plans for implementation.

## Conclusion

This qualitative content analysis provided insights into athletes’ experiences of using the mobile app BAck iN the Game (BANG) during rehabilitation following ACLR. The analysis of the interviews illustrates athletes’ awareness in interacting with, and the challenging and supportive experiences of using the app. The BANG app might provide support for returning to sport, primarily psychological support, as an adjunct to regular physiotherapy-guided rehabilitation. Combining the BANG app with usual care after ACLR might be an accommodating strategy in rehabilitation for people who wish to return to sport. Athletes’ experiences of the BANG app could be improved by healthcare professionals providing additional advice about when to use which content and why.

### Electronic supplementary material

Below is the link to the electronic supplementary material.

Supplementary Material 1

## Data Availability

Deidentified data is available from the last author, Joanna Kvist ( joanna.kvist@liu.se) upon reasonable request.

## Declarations

### Guidelines and regulations

We confirm that all methods were performed in accordance with the relevant guidelines and regulations.
