# Supplementary material for: Athletes’ experiences of using a self-directed psychological support, the BAck iN the Game (BANG) smartphone application, during rehabilitation for return to sports following anterior cruciate ligament reconstruction
Source: BMC Sports Sci Med Rehabil. 2023 Sep 19;15:113. doi: 10.1186/s13102-023-00731-2 (PMC10507891; doi:10.1186/s13102-023-00731-2)
Supplement: Supplementary file 1 — Supplementary Material 1 [file 13102_2023_731_MOESM1_ESM.docx]

Appendix 1

Interview guide

As far as I understand, you have had ACLR surgery *x* months ago and then you have had access to the app.

- Please tell me how your rehabilitation has been?
  - What was your preinjury sport?
  - What is your current activity level?
  - Are you done with your rehabilitation? Satisfied? What’s the plan from here?
- How did you experience using the app?
- What do you think of the app?
- Did you have any expectations before starting with the app? Were they fulfilled?
- The aim of the app was to provide psychological support to return to sport.
  - Do you think you’ve received psychological support? In what way?
  - Did you think that you would need psychological support to return to sport before you used the app?
  - Did you need psychological support during your rehabilitation?
  - Have you used any similar support before?
  - Did you receive any other effects from using the app? E.g., coping with stress? Other?
- Did you experience any barriers and/or facilitators for using the app?
- Content
  - Did you find some parts of the app more difficult and/or easier to grasp than others?
  - Please rank the content that was most helpful for you: videos, text, audio, questions about self-confidence, other.
  - Did you think the content was sufficient for your needs? Was there something missing?
- Usability
  - What did you think about the design of the app?
    - What did you think about the flow of the content? Was it easy to navigate the app?
    - How easy or difficult was it to remember to use the app/answer questions? How can it become more accessible, so you don’t forget to use it?
    - Do you have any suggestions how to make the content easier to navigate?
  - Would you recommend the app to others?
  - What was most annoying with the app?
- Do you think you will continue using the app?
- Any other comments?
